# Unraveling the Intraday Variations in the Tear Fluid Proteome

**DOI:** 10.1167/iovs.65.3.2

**Published:** 2024-03-05

**Authors:** Garrett Jones, Jeremy Altman, Saleh Ahmed, Tae Jin Lee, Wenbo Zhi, Shruti Sharma, Ashok Sharma

**Affiliations:** 1 Center for Biotechnology and Genomic Medicine, Medical College of Georgia, Augusta University, Augusta, GA, United States; 2 Department of Ophthalmology, Medical College of Georgia, Augusta University, Augusta, GA, United States; 3 Department of Population Health Sciences, Medical College of Georgia, Augusta University, Augusta, GA, United States

**Keywords:** tear fluid, proteome, biomarkers, mass spectrometry, intraday variations

## Abstract

**Purpose:**

Tear fluid is a complex and dynamic biological fluid that plays essential roles in maintaining ocular homeostasis and protecting against the external environment. Owing to the small sample volume, studying the tear proteome is challenging. However, advances in high-resolution mass spectrometry have expanded tear proteome profiling, revealing >500 unique proteins. Tears are emerging as a noninvasive source of biomarkers for both ocular and systemic diseases; nevertheless, intraday variability of proteins in tear fluid remains questionable. This study investigates intraday variations in the tear fluid proteome to identify stable proteins that could act as candidate biomarkers.

**Methods:**

Tear samples from 15 individuals at four time points (10 am, 12 pm, 2 pm, and 4 pm ) were analyzed using mass spectrometry to evaluate protein variation during these intervals. Technical variation was assessed by analyzing pooled samples and was subtracted from the total variation to isolate biological variability.

**Results:**

Owing to high technical variation, low-abundant proteins were filtered, and only 115 proteins met the criteria for further analysis. These criteria include being detected at all four time points in at least eight subjects, having a mean peptide-spectrum match count greater than 5, and having a technical variation less than 0.10. Lactotransferrin, lipocalin-1, and several immunoglobulins were among the 51 stable proteins (mean biological coefficient of variation < 0.10). Additionally, 43 proteins displayed significant slopes across the 4 time points, with 17 increasing and 26 decreasing over time.

**Conclusions:**

These findings contribute to the understanding of tear fluid dynamics and further expand our knowledge of the tear proteome.

Tear fluid contains an abundance of proteins that play vital roles in surface lubrication, ocular homeostasis, wound healing, and protection from the external environment.^1^^–^^4^ Previous studies have revealed that the majority of the tear-proteome content can be attributed to lactotransferrin, lipocalin-1, albumin, lysozyme C, and immunoglobulins. Continuous improvements in high-resolution workflows have led to significant advancements in tear proteome profiling.^5^^,^^6^ Notably, the reported human tear proteome has expanded from approximately 50 proteins in early discovery studies to now well over 500 unique proteins.^6^^–^^10^ As a result of these advancements, there has been a surge in the literature investigating the relationship between tear fluid proteins and ocular homeostasis, aging, and pharmacotherapy. These studies have reported changes in tear fluid proteomic composition in both ocular and systemic pathological conditions.^11^^–^^19^ Thus, the determination of tear protein levels is gaining significance as a potential source of biomarkers.

However, like other biological fluids, tear film experiences normal fluctuations in its content. Consequently, there is a growing need for a comprehensive understanding of physiological variations, including intraday deviations, to enable more accurate comparisons of tear protein levels across different studies and disease states. Analyzing the intraday variation in the tear fluid proteome provides valuable insights for assessing the suitability of specific tear proteins as biomarkers and brings tear fluid profiling closer to clinical application. This advancement is particularly beneficial for individuals affected by ocular surface diseases, such as dry eye, who currently face challenges in diagnosis and have limited treatment options.^20^

By examining changes in tear composition throughout the day, we can identify proteins that maintain stable levels, making them suitable biomarker candidates. This approach helps to decrease the impact of daily fluctuations, ensuring more reliable results in biomarker panels. As a result, the reproducibility of findings and data sharing in the field of tear proteomics will significantly improve, especially considering the impracticality of long-term control over the time of collection. Thus, in this study, we conducted a comprehensive investigation into intraday variability in the tear fluid proteome using our previously established mass spectrometry–based workflow for proteomic analysis of tear fluid.^9^

## Materials and Methods

### Subjects

This study was approved by the Institutional Review Board at Augusta University (IRB Project ID# 1458143), and written informed consent was obtained from all study participants. Tear samples were collected from 15 healthy participants (8 males, 7 females), ranging from 23 to 54 years of age. The participants were asked to confirm the absence of any preexisting ocular conditions, acute illnesses, chronic autoimmune disorders, current use of contact lenses, recent ocular surgeries within the last 2 months, or application of topical medications within the preceding 24 hours, thus minimizing the influence of potential confounding variables. Additionally, each participant completed the Ocular Surface Disease Index questionnaire to assess symptoms of ocular irritation before sample collection. Participants reporting an Ocular Surface Disease Index score of >12 were excluded. The Ocular Surface Disease Index score, Schirmer strip wetting length, and other relevant subject information are displayed in Table 1.

**Table 1.** Demographic and Sample Information of Participants

				Wetted Length (mm)				
Subject	Sex	Age (Years)	Ocular Surface Disease Index Score	10 am	12 pm	2 pm	4 pm	Eye
S01	Male	24	6.25	9	8	19	17	OD
S02	Female	23	0	3	9	7	5	OS
S03	Female	28	4.17	17	13	13	15	OD
S04	Male	33	0	6	3	4	6	OS
S05	Male	23	10.42	12	20	11	12	OD
S06	Male	48	4.17	20	18	15	13	OD
S07	Female	32	0	18	5	9	15	OS
S08	Male	54	0	17	15	19	23	OS
S09	Female	53	0	30	30	30	30	OD
S10	Male	27	2.08	11	7	2	7	OS
S11	Female	30	10.42	30	30	30	16	OD
S12	Male	28	0	10	4	7	5	OD
S13	Female	32	0	10	9	5	7	OD
S14	Male	53	8.33	5	25	23	14	OS
S15	Female	53	4.17	6	26	8	3	OD

### Sample Collection

Tear samples were collected using Schirmer strips (TearFlo, HUB Pharmaceuticals, Scottsdale, AZ, USA) without the use of topical anesthesia. Sampling was conducted between 10:00 am and 4:00 pm to reflect common clinic hours. Each day, one participant provided tear samples at 10:00 am, 12:00 pm, 2:00 pm, and 4:00 pm. The collection protocol is as follows: a Schirmer strip was folded at the 0-mm mark (within the sterile package), removed with a gloved hand, and inserted into the lateral portion of the lower eyelid for 5 minutes. To ensure comfort and consistency, the subjects' eyes remained closed during the collection process. Samples were taken from the same eye at all four time points. Upon removal, the saturated strip was transferred immediately into a 1.5-mL vial (#05408129, Thermo Fisher Scientific, Waltham, MA, USA), placed on dry ice, and transferred to a −80°C freezer for storage.

### Protein Extraction and Digestion

For protein extraction and digestion, we used the in-strip protein digestion method, as previously published.^9^ In this method, Schirmer strips were initially lyophilized and then cut into pieces measuring 5.0 mm × 2.5 mm. To denature the proteins, 120 µL of 8 M urea in 50 mM Tris-HCl (pH 8) was added. After this, samples were reduced with 10 mM dithiothreitol and alkylated with 55 mM iodoacetamide. The pH of each sample was adjusted within the range of 7 to 9 using 0–14 pH strips (#13640516, Thermo Fisher Scientific) before subjecting them to digestion with mass spectrometry-graded trypsin (#90057, Thermo Fisher Scientific) at a 1:20 trypsin to protein (w/w) ratio. This digestion process occurred overnight at 37°C.

Upon completion of digestion, the peptide concentration was determined using the Pierce Quantitative Colorimetric Peptide Assay (#23275, Thermo Fisher Scientific) to ensure the appropriate concentration for each sample before proceeding to the subsequent step. The protein concentration of each digested sample ranged from 200 to 250 µg/mL. For further processing, an aliquot containing 125 µg of protein was extracted from each sample. Additionally, 125 µL from each of the 60 digested samples, irrespective of concentration, was pooled to assess technical variation.

The digested peptides were further purified using C18 spin columns and subsequently lyophilized. The purified peptides were then reconstituted with 80 µL of equilibration buffer (2% acetonitrile in 0.1% formic acid) in preparation for analysis using an Orbitrap Fusion Tribrid mass spectrometer (Thermo Fisher Scientific) in conjunction with the Ultimate 3000 nano-UPLC system (#744101, Harvard Apparatus, Holliston, MA, USA).

### Liquid Chromatography-Tandem Mass Spectrometry

Four microliters of reconstituted peptides were loaded and washed on a Pepmap100 C18 trap (5 µm, 0.3 × 5.0 mm, Thermo Fisher Scientific, Waltham, MA, USA) at a flow rate of 20 µL/min. The washing process was carried out using 2% acetonitrile in water (with 0.1% formic acid) for 10 minutes. Subsequently, the peptides were separated on a Pepmap100 RSLC C18 column (2.0 µm, 75 µm × 150 mm, Thermo Fisher Scientific) using a multistep gradient of 2% to 40% acetonitrile with 0.1% formic acid over a period of 150 minutes. The separation was achieved at a flow rate of 300 nL/min and a column temperature of 40°C, allowing for efficient chromatographic resolution. To analyze the eluted peptides, we used the Orbitrap Fusion mass spectrometry instrument with a nano-electrospray ionization source, set at a temperature of 300°C and a spray voltage of 2000 V. The instrument operated in data-dependent acquisition mode in positive polarity. During the precursor scan, the Orbitrap mass spectrometry analyzer was used with a resolution of 120,000 fill-width half-maximum, encompassing the m/z range of 400 to 2000. An ion-trap mass spectrometry analyzer was used for tandem mass spectrometry scans in top speed mode, with dynamic exclusion settings of repeat count of 1, repeat duration of 15 seconds, exclusion duration of 30 seconds, and a cycle time of 3 seconds.

### Protein Identification and Analysis

The raw mass spectrometry data were processed using the Proteome Discoverer software (version 1.4, Thermo Fisher Scientific). A SequestHT search against the SwissProt human database was performed and search parameters included a precursor tolerance of 10 ppm and a product ion tolerance of 0.6 Da. For accurate identification, specific modifications were considered during the search. Static carbidomethylation (+57.021 Da) was applied to cysteine residues, while dynamic oxidation (+15.995 Da) was applied to methionine residues. Peptide-spectrum match (PSM) validation was carried out using the percolator algorithm to ensure reliable identifications. Some proteins could not be distinguished unambiguously solely based on the database search outcomes. In such cases, proteins were clustered together according to the principles of parsimony to minimize redundancy in the final results. A comprehensive report was generated, providing detailed information about the identities and PSM count for each protein. The PSM counts were used as a semiquantitative measure, enabling us to gain insights into the relative abundance of proteins in the tear fluid proteome.

### Assessment of Technical Variation

To assess technical variation, the pooled mixture containing 125 µL from each of the 60 digested samples was divided into 15 identical aliquots. Each aliquot was processed and analyzed using identical steps as previously described. For each of the proteins, the data were log transformed, four samples were randomly selected, and the corresponding coefficient of variation (CV) was calculated to mimic the protein's CV for an individual subject. This was repeated 15 times to calculate an average technical CV. To increase the statistical reliability, the procedure of randomly selecting four samples and calculating the average CV was repeated 30 times for each protein. The results were then averaged to determine the technical variation associated with each of the identified proteins. Finally, the technical variation CV was subtracted from the overall CV calculated from the nonpooled samples to isolate the biological variation.

### Statistical Analysis

All statistical analyses were conducted using R (version 4.3.1; The R Foundation for Statistical Computing, Vienna, Austria), with a significance level set at *P* value of less than 0.05. Proteomic data underwent initial descriptive and graphical assessments, and data quality control measures were implemented before progressing to hypothesis testing and statistical modeling. To gauge the consistency of protein levels over time, we computed the CV. Technical variabilities for each protein were computed as outlined in the earlier section. The technical CV was subtracted from the total CV to derive the final values of biological CV between samples collected across four time points.

For evaluation of time-dependent trends, a regression line was fitted for each individual subject, and the average slope for each protein was calculated. From this, the significance of the time effect was assessed using a *t* test.

## Results

### Proteomic Profiling of Tear Samples

**Figure 1.**
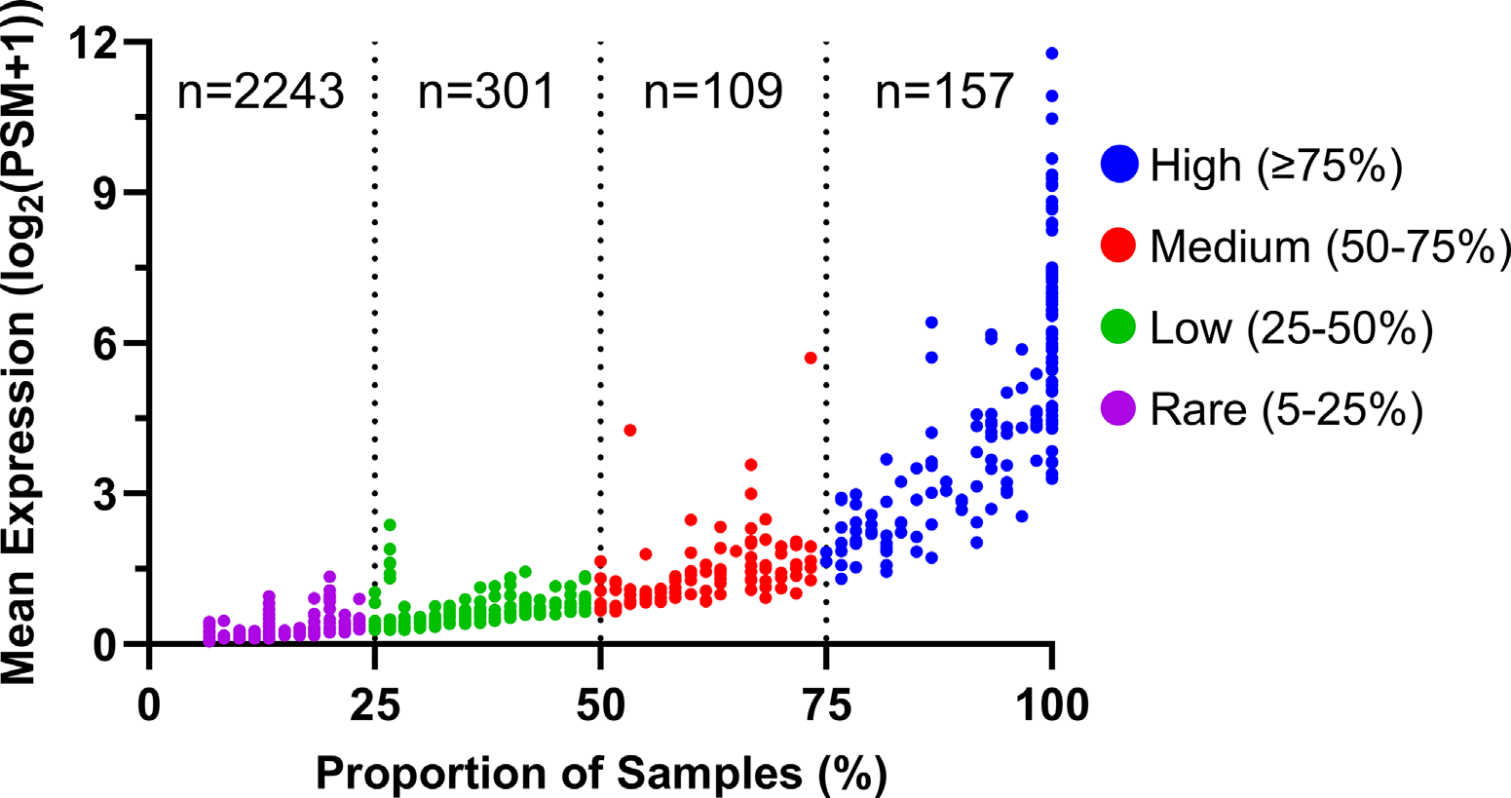
Distribution of protein levels detected in 60 human tear samples with PSM ≥2 and detection in >5% of samples. The *x*-axis portrays the detection frequency of each protein, and the *y*-axis portrays the average protein levels (quantified by PSMs). These proteins were classified based on their abundance into four groups: high abundance (157 proteins, detected in ≥75% of samples), medium (109 proteins, 50%–75% of samples), low (301 proteins, 25%–50% of samples), and rare (2243 proteins, 5%–25% of samples).

A total of 60 tear fluid samples (15 subjects, 4 time points each) were analyzed using mass spectrometry, and 2810 unique proteins were detected. However, in mass spectrometry, owing to the stochastic nature of ionization and detection processes, the low-abundance proteins face greater challenges in being detected consistently and reliably. In some samples, these proteins may be detected, whereas in others, they may go undetected. This variability poses a challenge in achieving reproducibility and reliability when studying proteins present at low concentrations. For this reason, we excluded low-abundance proteins when assessing intraday variations. The average protein levels (quantified by the number of PSMs) and the proportion of samples in which each protein was detected are shown in Figure 1. Based on their relative abundance, these proteins were categorized into four groups: high abundance (detected in ≥75% of samples), medium (50%–75% of samples), low (25%–50% of samples), and rare (5%–25% of samples). Notably, 266 proteins were detected in ≥50% of the samples analyzed. The 20 most abundant proteins detected in tear fluid are listed in Table 2.

**Table 2.** Top 20 Most Abundant Proteins Detected in Tear Fluid

Protein ID	Description	Gene Name	Average PSM	Detected in Proportion of Samples (%)	Total CV	Biological CV
P02788	Lactotransferrin	LTF	3495.89	100	0.040	0.032
P02768	Albumin	ALB	1949.43	100	0.056	0.047
P31025	Lipocalin-1	LCN1	1419.50	100	0.042	0.027
P01876	Immunoglobulin heavy constant alpha 1	IGHA1	816.30	100	0.050	0.040
P61626	Lysozyme C	LYZ	652.18	100	0.053	0.043
P01833	Polymeric immunoglobulin receptor	PIGR	624.76	100	0.056	0.045
P12273	Prolactin-inducible protein	PIP	575.23	100	0.057	0.047
P01834	Immunoglobulin kappa constant	IGKC	560.55	100	0.048	0.039
P0DOX7	Immunoglobulin kappa light chain	IGK	452.94	100	0.051	0.042
P25311	Zinc-alpha-2-glycoprotein	AZGP1	422.28	100	0.060	0.051
P0DOX2	Immunoglobulin alpha-2 heavy chain	IGHA2	405.13	100	0.058	0.044
Q9GZZ8	Extracellular glycoprotein lacritin	LACRT	336.38	100	0.057	0.046
P01036	Cystatin-S	CST4	328.10	100	0.068	0.054
O75556	Mammaglobin-B	SCGB2A1	302.99	100	0.061	0.048
P0DOY2	Immunoglobulin lambda constant 2	IGLC2	180.48	100	0.067	0.054
Q99935	Opiorphin prepropeptide	OPRPN	173.30	100	0.070	0.061
Q16378	Proline-rich protein 4	PRR4	169.37	100	0.071	0.035
P02787	Serotransferrin	TF	161.88	100	0.129	0.121
P60709	Actin, cytoplasmic 1	ACTB	161.80	100	0.067	0.046
P01037	Cystatin-SN	CST1	154.65	100	0.092	0.076

### Assessment of Technical and Biological Variation in Tear Protein Levels Over Time

**Figure 2.**
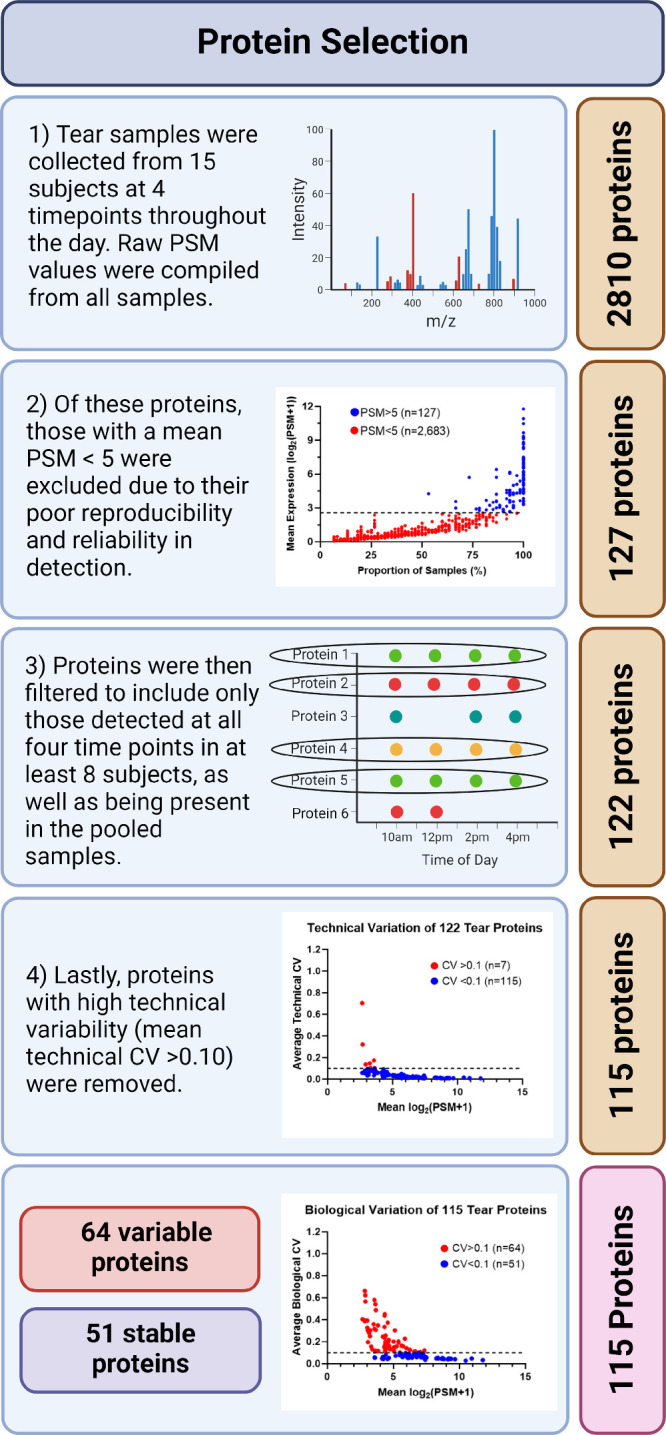
Workflow for protein selection. The initial filter was used to remove low-abundant proteins (with a mean PSM count of < 5) resulting in 127 proteins. Proteins were then filtered to include only those detected at all four time points in a minimum of eight subjects as well as in the pooled sample (122 proteins). Last, proteins with high technical variability (mean technical CV > 0.10) were excluded, resulting in 115 proteins.

For an accurate assessment of the variation in tear protein levels over time, we implemented several filters during the data analysis stage (Fig. 2). First, proteins present at very low concentrations (mean PSM of <5) were excluded owing to their poor reproducibility and reliability in detection, resulting in 127 proteins. Next, we ensured that we had an adequate number of data points to assess both intraday and technical variations. Thus, we filtered our data to include only proteins detected at all 4 time points in at least 8 of the 15 subjects, and we excluded proteins that were not present in the pooled samples. This process resulted in 122 proteins. Finally, proteins exhibiting high technical variability (mean technical CV of >0.10) were excluded from further analyses. These stringent filters led to a total of 115 proteins that were considered for the rest of our analysis (Supplementary Table S1).

**Figure 3.**
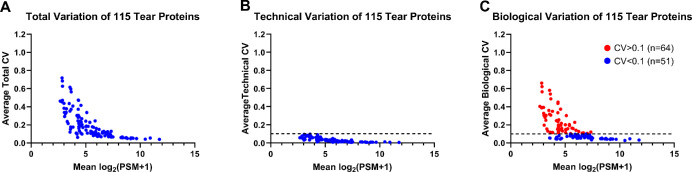
Variation in expression of 115 tear proteins. (**A**) Calculated average total CV of 115 proteins, (**B**) technical variation of the 115 proteins determined via technical replicates of pooled tear samples, and (**C**) biological variation of the 115 proteins obtained by subtracting the technical variability from the total variability.

Subsequently, we subtracted the technical variability from the total CV to derive a more accurate representation of the biological variability of each of the remaining 115 tear proteins (Fig. 3). This analytical approach allowed us to identify proteins with consistent expression trends throughout the day, as well as those exhibiting significant changes in expression over time. Among the 115 proteins analyzed, 51 proteins displayed a biological variability of less than 10% across the four time points, indicating a high level of consistency. In contrast, 64 proteins exhibited an average biological CV of more than 10% over the course of the day, indicating notable variability in their expression levels.

### Tear Proteins With Minimal Intraday Variability

**Figure 4.**
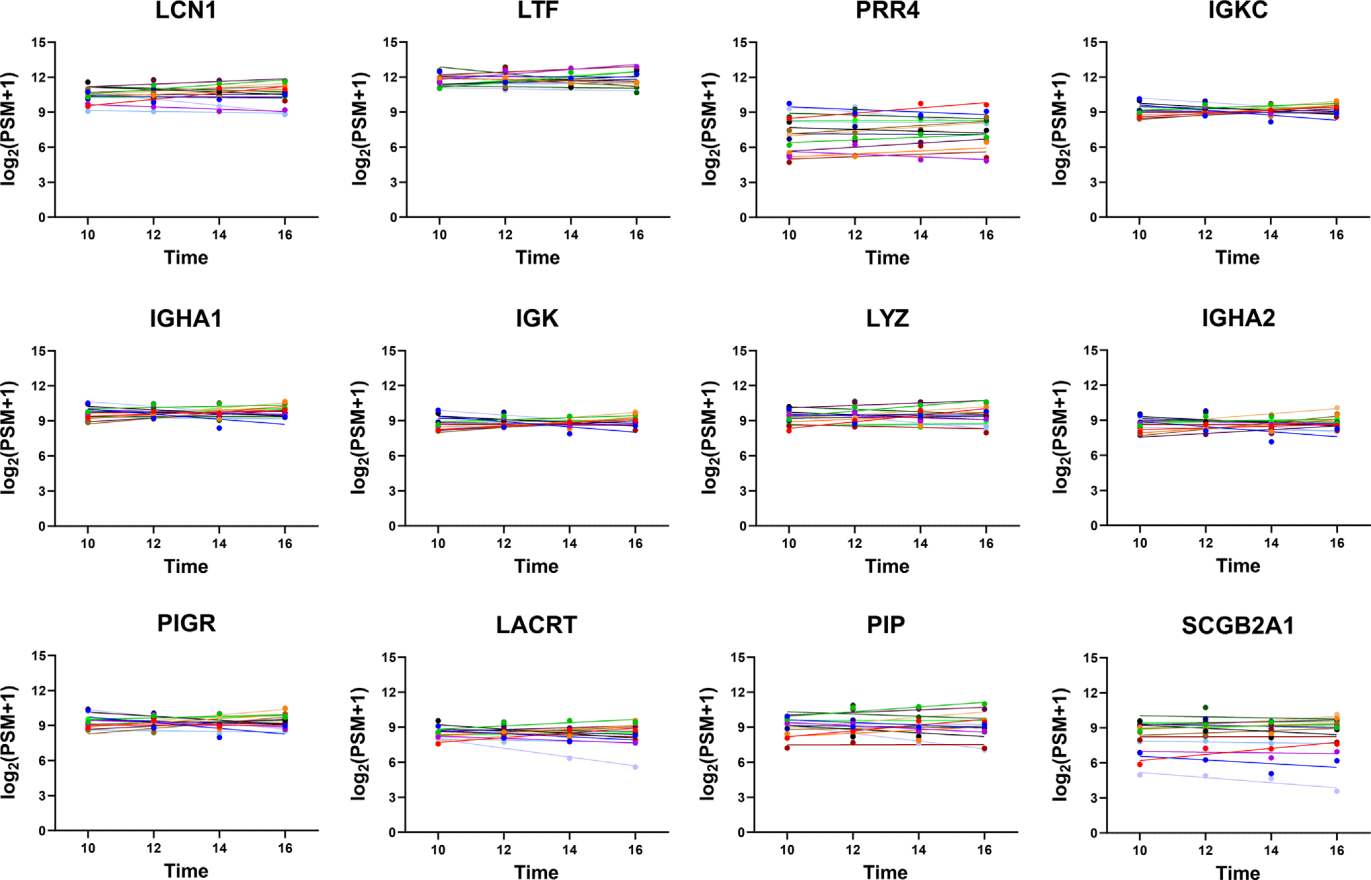
Top 12 proteins with lowest intraday variation. The *x*-axis portrays the sample collection time, and the *y*-axis portrays the protein levels (quantified by PSMs). Individual subjects are color coded and displayed with their line of best fit.

A total of 51 proteins had an average biological CV of less than 10%. Representative plots of the top 12 most stable proteins, depicting their consistent trends over time, are shown in Figure 4. The most stable proteins in tear fluid include lipocalin-1, lactotransferrin, proline-rich protein 4, immunoglobulin kappa constant, immunoglobulin heavy constant alpha 1, immunoglobulin kappa light chain, lysozyme C, immunoglobulin alpha-2 heavy chain, polymeric immunoglobulin receptor, extracellular glycoprotein lacritin, prolactin-inducible protein, and mammaglobin-B. As expected, overall protein levels demonstrate modest interpersonal variability; however, the levels at four different time points in a particular subject are similar. For a comprehensive view, the overall CV, technical CV, biological CV, and slope value of the 31 stable proteins with a |slope| of less than 0.05 are listed in Table 3. The additional proteins and their CVs are provided in Supplemental Table S1.

**Table 3.** The 31 Most Stable Tear Proteins With Biological CV < 0.10 and |Slope| < 0.05

Protein ID	Description	Gene Name	Total CV	Biological CV	Slope	*P* Value
P31025	Lipocalin-1	LCN1	0.042	0.027	0.023	0.53
P02788	Lactotransferrin	LTF	0.040	0.032	0.014	0.70
Q16378	Proline-rich protein 4	PRR4	0.071	0.035	0.032	0.40
P01834	Immunoglobulin kappa constant	IGKC	0.048	0.039	0.023	0.53
P01876	Immunoglobulin heavy constant alpha 1	IGHA1	0.050	0.040	0.021	0.59
P0DOX7	Immunoglobulin kappa light chain	IGK	0.051	0.042	0.020	0.59
P61626	Lysozyme C	LYZ	0.053	0.043	0.029	0.43
P0DOX2	Immunoglobulin alpha-2 heavy chain	IGHA2	0.058	0.044	0.014	0.73
P01833	Polymeric immunoglobulin receptor	PIGR	0.056	0.045	0.002	0.95
Q9GZZ8	Extracellular glycoprotein lacritin	LACRT	0.057	0.046	−0.006	0.89
P12273	Prolactin-inducible protein	PIP	0.057	0.047	0.007	0.87
O75556	Mammaglobin-B	SCGB2A1	0.061	0.048	0.004	0.89
Q5VSP4	Putative lipocalin 1-like protein 1	LCN1P1	0.062	0.048	−0.011	0.88
P25311	Zinc-alpha-2-glycoprotein	AZGP1	0.060	0.051	−0.006	0.88
P68032	Actin, alpha cardiac muscle 1	ACTC1	0.072	0.051	−0.037	0.22
P03973	Antileukoproteinase	SLPI	0.100	0.051	0.013	0.74
B9A064	Immunoglobulin lambda-like polypeptide 5	IGLL5	0.070	0.053	0.036	0.45
P01036	Cystatin-S	CST4	0.068	0.054	−0.019	0.71
A0A075B6K5	Immunoglobulin lambda variable 3-9	IGLV3-9	0.152	0.054	0.033	0.41
P0DOY2	Immunoglobulin lambda constant 2	IGLC2	0.067	0.054	0.038	0.37
Q9UGM3	Deleted in malignant brain tumors 1 protein	DMBT1	0.069	0.057	0.002	0.95
P01591	Immunoglobulin J chain	JCHAIN	0.081	0.059	0.011	0.81
Q99935	Opiorphin prepropeptide	OPRPN	0.070	0.061	0.027	0.54
P01619	Immunoglobulin kappa variable 3-20	IGKV3-20	0.098	0.064	0.005	0.90
P10909	Clusterin	CLU	0.088	0.071	−0.008	0.81
P01037	Cystatin-SN	CST1	0.092	0.076	−0.020	0.74
P13645	Keratin, type I cytoskeletal 10	KRT10	0.098	0.076	−0.011	0.81
P14555	Phospholipase A2, membrane associated	PLA2G2A	0.134	0.081	0.020	0.48
P04264	Keratin, type II cytoskeletal 1	KRT1	0.100	0.089	−0.036	0.44
P09228	Cystatin-SA	CST2	0.103	0.091	0.006	0.93
P35527	Keratin, type I cytoskeletal 9	KRT9	0.127	0.096	−0.022	0.69

### Intraday Variability of Tear Protein Levels Reveals Time-dependent Trends

**Figure 5.**
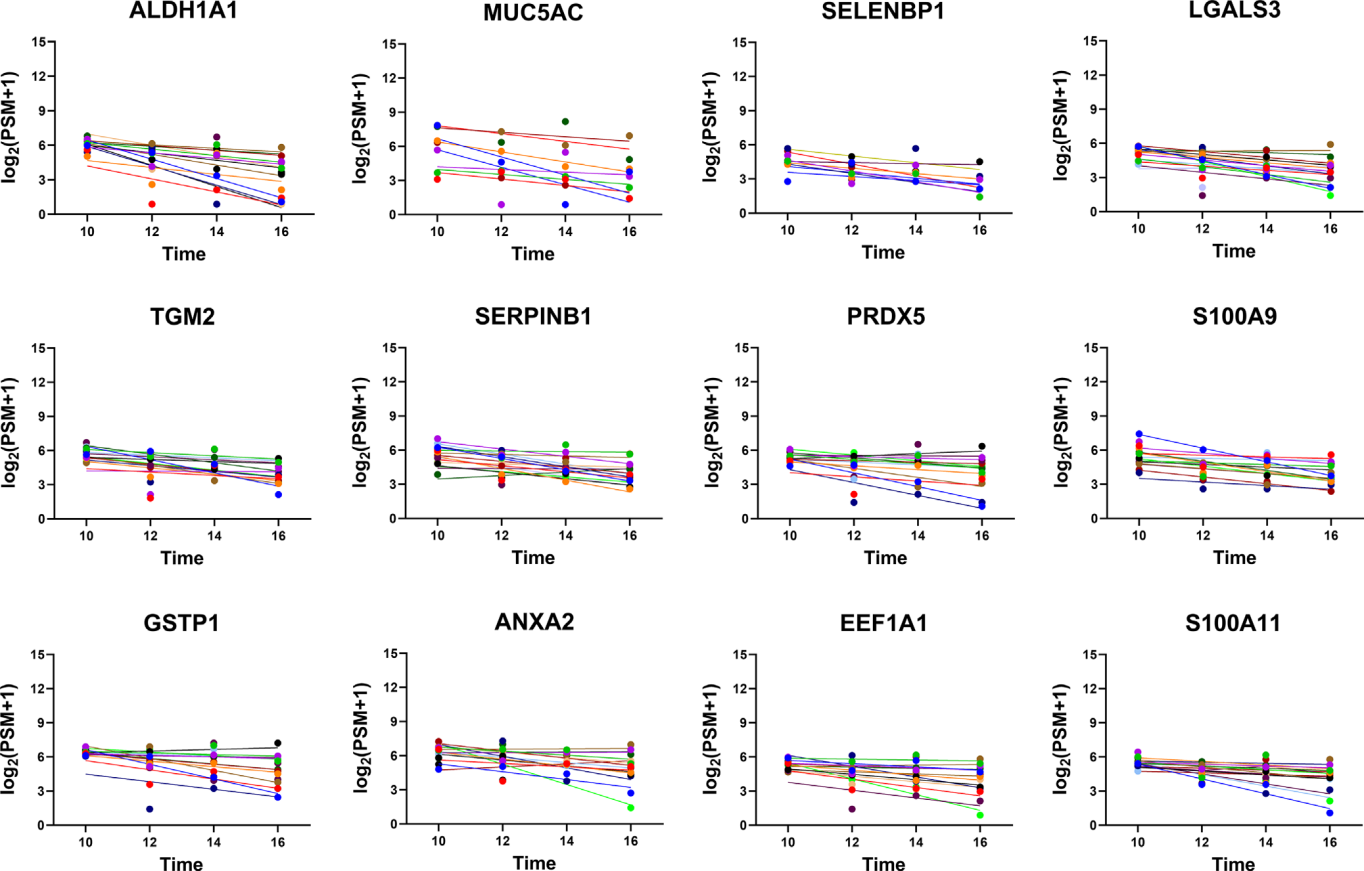
Top 12 proteins with the most negative slopes. The *x*-axis portrays the sample collection time, and the *y*-axis portrays the protein levels (quantified by PSMs). Individual subjects are color coded and displayed with their line of best fit.

**Figure 6.**
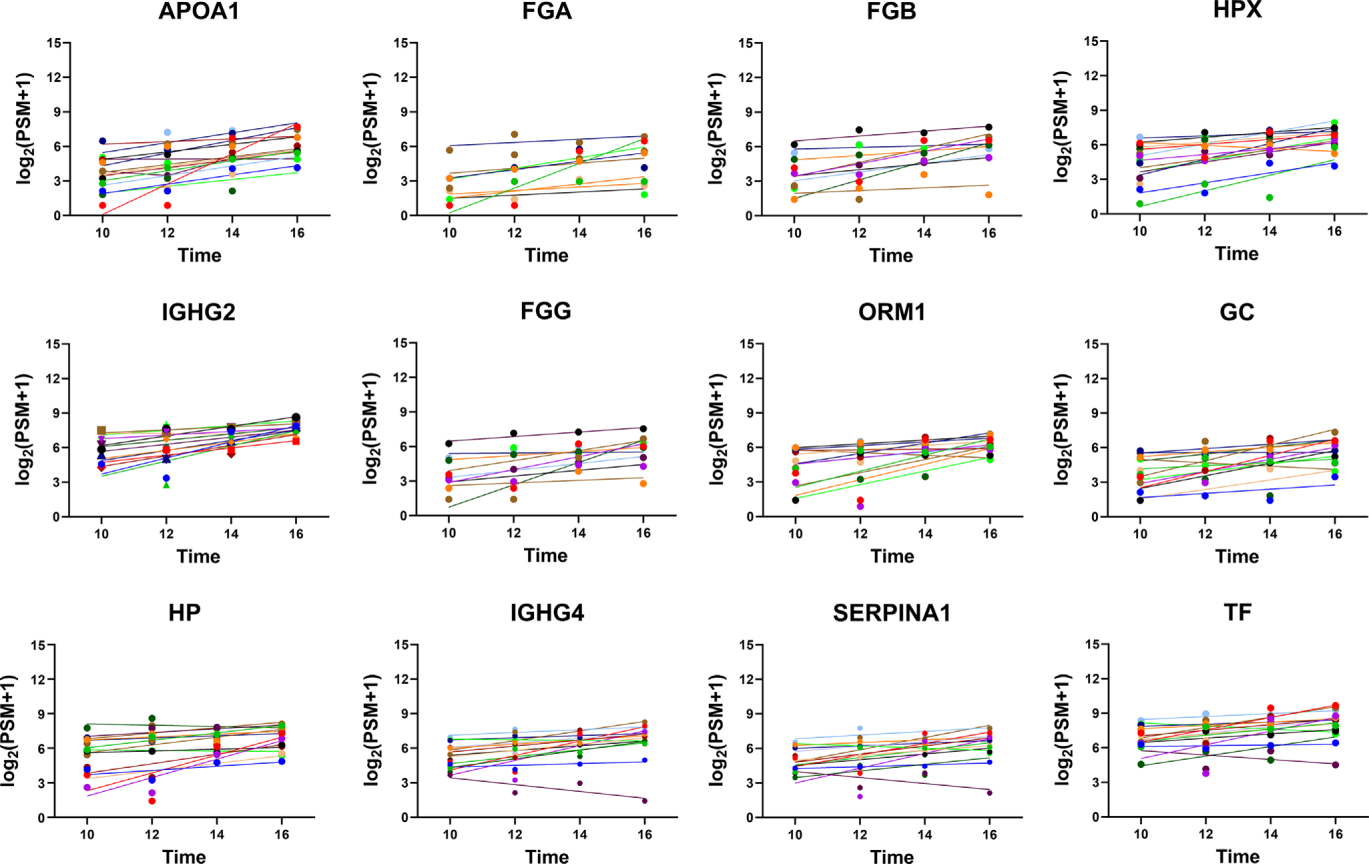
Top 12 proteins with greatest positive slopes. The *x*-axis portrays the sample collection time, and the *y*-axis portrays the protein levels (quantified by PSMs). Individual subjects are color-coded and displayed with their line of best fit.

To examine the intraday trend in tear protein levels, we plotted the protein levels at all four time points (10 am, 12 pm, 2 pm, and 4 pm) for each individual protein. Using linear regression analysis, we calculated the slope for each protein to measure the deviation in protein levels over time. The results of our analysis revealed intriguing patterns in tear protein dynamics. Out of the 64 proteins with a biological CV of more than 0.10, 43 proteins exhibited a significant slope. Among these 43 proteins, 26 decreased (slope of <−0.10) (Table 4), whereas 17 increased (slope of >0.10) (Table 5) between 10 am and 4 pm. The representative proteins with the most negative slope and positive slope are shown in Figure 5 and Figure 6, respectively. A detailed listing of these proteins and their corresponding trends, including comprehensive information on the identified proteins with intraday variability, is provided in Supplementary Table S1.

**Table 4.** The 26 Variable Proteins With Negative Slopes (Decreasing Trend Over Time)

Protein ID	Description	Gene Name	Total CV	Biological CV	Slope	*P* Value
P00352	Retinal dehydrogenase 1	ALDH1A1	0.474	0.437	−0.446	6.5 × 10^−5^
P98088	Mucin-5AC	MUC5AC	0.616	0.582	−0.396	3.4 × 10^−3^
Q13228	Methanethiol oxidase	SELENBP1	0.719	0.662	−0.303	9.4 × 10^−4^
P17931	Galectin-3	LGALS3	0.244	0.152	−0.257	1.4 × 10^−4^
P21980	Protein-glutamine gamma-glutamyltransferase 2	TGM2	0.269	0.218	−0.253	4.1 × 10^−5^
P30740	Leukocyte elastase inhibitor	SERPINB1	0.202	0.162	−0.248	3.4 × 10^−4^
P30044	Peroxiredoxin-5, mitochondrial	PRDX5	0.287	0.257	−0.246	1.8 × 10^−3^
P06702	Protein S100-A9	S100A9	0.240	0.197	−0.246	4.4 × 10^−5^
P09211	Glutathione S-transferase P	GSTP1	0.230	0.203	−0.245	6.6 × 10^−4^
P07355	Annexin A2	ANXA2	0.227	0.192	−0.233	5.0 × 10^−3^
P68104	Elongation factor 1-alpha 1	EEF1A1	0.240	0.196	−0.218	1.2 × 10^−3^
P31949	Protein S100-A11	S100A11	0.231	0.196	−0.217	6.9 × 10^−4^
P00338	L-lactate dehydrogenase A chain	LDHA	0.435	0.360	−0.211	4.9 × 10^−3^
P14618	Pyruvate kinase PKM	PKM	0.184	0.158	−0.208	3.4 × 10^−3^
P26447	Protein S100-A4	S100A4	0.172	0.132	−0.201	2.6 × 10^−4^
Q06830	Peroxiredoxin-1	PRDX1	0.184	0.149	−0.198	3.2 × 10^−3^
P00558	Phosphoglycerate kinase 1	PGK1	0.627	0.565	−0.192	1.0 × 10^−2^
P30041	Peroxiredoxin-6	PRDX6	0.216	0.131	−0.190	3.1 × 10^−5^
P62937	Peptidyl-prolyl cis-trans isomerase A	PPIA	0.353	0.318	−0.173	2.4 × 10^−2^
P30086	Phosphatidylethanolamine-binding protein 1	PEBP1	0.453	0.388	−0.162	1.5 × 10^−3^
P23528	Cofilin-1	CFL1	0.238	0.156	−0.160	8.5 × 10^−4^
P60174	Triosephosphate isomerase	TPI1	0.177	0.132	−0.154	7.3 × 10^−3^
P04075	Fructose-bisphosphate aldolase A	ALDOA	0.338	0.250	−0.143	1.1 × 10^−3^
P0DMV8	Heat shock 70 kDa protein 1A	HSPA1A	0.144	0.115	−0.126	2.6 × 10^−2^
P37802	Transgelin-2	TAGLN2	0.372	0.307	−0.125	4.0 × 10^−2^
P80188	Neutrophil gelatinase-associated lipocalin	LCN2	0.161	0.113	−0.117	3.4 × 10^−3^

**Table 5.** The 17 Variable Proteins With Positive Slopes (Increasing Trend Over Time)

Protein ID	Description	Gene Name	Total CV	Biological CV	Slope	*P* Value
P02647	Apolipoprotein A-I	APOA1	0.471	0.451	0.410	4.2 × 10^−4^
P02671	Fibrinogen alpha chain	FGA	0.685	0.623	0.358	1.4 × 10^−2^
P02675	Fibrinogen beta chain	FGB	0.565	0.487	0.356	2.7 × 10^−3^
P02790	Hemopexin	HPX	0.336	0.303	0.344	1.6 × 10^−4^
P01859	Immunoglobulin heavy constant gamma 2	IGHG2	0.173	0.159	0.338	6.6 × 10^−5^
P02679	Fibrinogen gamma chain	FGG	0.596	0.540	0.336	8.0 × 10^−3^
P02763	Alpha-1-acid glycoprotein 1	ORM1	0.415	0.374	0.329	1.3 × 10^−3^
P02774	Vitamin D-binding protein	GC	0.372	0.335	0.314	8.0 × 10^−4^
P00738	Haptoglobin	HP	0.245	0.227	0.270	1.8 × 10^−3^
P01861	Immunoglobulin heavy constant gamma 4	IGHG4	0.160	0.140	0.229	2.3 × 10^−3^
P01009	Alpha-1-antitrypsin	SERPINA1	0.179	0.163	0.228	2.0 × 10^−3^
P02787	Serotransferrin	TF	0.129	0.121	0.219	2.0 × 10^−3^
P13647	Keratin, type II cytoskeletal 5	KRT5	0.125	0.107	0.196	3.2 × 10^−3^
P01860	Immunoglobulin heavy constant gamma 3	IGHG3	0.123	0.109	0.193	2.5 × 10^−3^
P02538	Keratin, type II cytoskeletal 6A	KRT6A	0.149	0.127	0.186	7.0 × 10^−3^
P62805	Histone H4	H4C1	0.409	0.349	0.145	3.3 × 10^−2^
P01700	Immunoglobulin lambda variable 1-47	IGLV1-47	0.293	0.197	0.116	2.5 × 10^−3^

## Discussion

Tear fluid is emerging as a promising source of biomarkers with the potential to advance the diagnosis of both systemic and ocular diseases using a noninvasive technique. Its composition has revealed a complex array of proteins that hold crucial information about the health of an individual. Although tear fluid offers an exciting avenue for biomarker discovery, the variability exhibited by tear proteins presents a central challenge that must be addressed. The intricate interplay of factors, such as the tear production rate, environmental influences, and intraday variations, has rendered the analysis and interpretation of tear biomarkers a challenging task. Cutting-edge techniques, including advanced mass spectrometry proteomic analyses and high-throughput multiplex immunoassays, are now available to unravel the complexity and harness the diagnostic potential of tear fluid. Mass spectrometry analysis provides numerous benefits compared with other proteomic methods, including an expanded dynamic range and enhanced capability to detect a wide range of proteins in very small quantities within tear samples.^21^^,^^22^ This result is evident from the rapid increase in unique proteins detected in tears.

Previous studies exploring intraday variations in tear film proteins have found minimal changes. However, these studies have focused primarily on total protein content or specific tear proteins, including albumin, lysozyme, transferrin, lacritin, epidermal growth factor, and immunoglobulin A.^23^^–^^30^ Albumin was reported to have higher expression towards the end of the day.^24^ Although our study found minimal intraday variation in albumin (biological CV = 0.047), it did have a significantly positive slope (0.152). A recent study examined tear protein content over a seven-day period and reported no significant interday differences in proteins from tears collected between 1:00 pm and 2:00 pm.^31^ This study also found no significant variability in tear peptide or protein profiles between the right and left eyes of the same individuals over this period. Another study investigated the intraday variation of tear cytokines by comparing their expression at midday and in the evening.^32^ It was observed that tear cytokine levels were generally higher in the evening compared with the earlier hours.

To the best of our knowledge, our study represents the first effort to investigate intraday protein variations in tears across a significant number of proteins. The highly abundant proteins, such as lysozyme C, lactotransferrin, lipocalin-1, immunoglobulin heavy constant alpha 1, and prolactin-inducible protein, are consistent with findings from previous studies.^7^^–^^9^^,^^33^^–^^39^ Despite detecting a large number of unique proteins, our ability to measure the CV extends to a specific subset of proteins (only 115 proteins were included in the analysis). This limitation arises from the inherent constraints within mass spectrometry, as we encounter challenges in accurately assessing variations in proteins present in low abundance. We observed high variability within technical replicates for these less abundant proteins, which hinders our capacity to precisely quantify and discern fluctuations in the levels over time. Further targeted studies are needed to reliably capture variations in other subsets of proteins, particularly for those present in limited quantities.

## Conclusions

Tear fluid has emerged as a valuable noninvasive source of biomarkers for the diagnosis and management of both ocular and systemic diseases. However, to move the field forward it is necessary to identify the temporal and diurnal variations of tear proteins. In this study, we successfully measured the intraday variation of 115 proteins in tear fluid. Of these, 51 proteins demonstrated intraday stability (mean biological CV of <0.10), a crucial attribute for effective biomarkers. Furthermore, 43 proteins exhibited notable trends across time, with 17 increasing and 26 decreasing over the 6-hour window during which the samples were collected. Only 115 highly abundant proteins were included in our analysis owing to the inherent constraints of shotgun mass spectrometry. Further targeted studies are needed to explore variations in other tear proteins not covered in this study.

## Supplementary Material

Supplement 1
